# Direct Binding of Bovine IgG-Containing Immune Complexes to Human Monocytes and Their Putative Role in Innate Immune Training

**DOI:** 10.3390/nu14214452

**Published:** 2022-10-22

**Authors:** Mojtaba Porbahaie, Huub F. J. Savelkoul, Cornelis A. M. de Haan, Malgorzata Teodorowicz, R. J. Joost van Neerven

**Affiliations:** 1 Cell Biology and Immunology, Wageningen University & Research, 6708 WD Wageningen, The Netherlands; 2 Virology Division, Infectious Diseases and Immunology, Utrecht University, 3584 CS Utrecht, The Netherlands; 3 FrieslandCampina, 3818 LE Amersfoort, The Netherlands

**Keywords:** immune complex, trained immunity, RSV, bovine IgG, preF protein

## Abstract

Bovine milk IgG (bIgG) was shown to bind to and neutralize the human respiratory synovial virus (RSV). In animal models, adding bIgG prevented experimental RSV infection and increased the number of activated T cells. This enhanced activation of RSV-specific T cells may be explained by receptor-mediated uptake and antigen presentation after binding of bIgG-RSV immune complexes (ICs) with FcγRs (primarily CD32) on human immune cells. This indirect effect of bIgG ICs on activation of RSV-specific T cells was confirmed previously in human T cell cultures. However, the direct binding of ICs to antigen-presenting cells has not been addressed. As bovine IgG can induce innate immune training, we hypothesized that this effect could be caused more efficiently by ICs. Therefore, we characterized the expression of CD16, CD32, and CD64 on (peripheral blood mononuclear cells (PBMCs), determined the optimal conditions to form ICs of bIgG with the RSV preF protein, and demonstrated the direct binding of these ICs to human CD14^+^ monocytes. Similarly, bIgG complexed with a murine anti-bIgG mAb also bound efficiently to the monocytes. To evaluate whether the ICs could induce innate immune training more efficiently than bIgG itself, the resulted ICs, as well as bIgG, were used in an in vitro innate immune training model. Training with the ICs containing bIgG and RSV preF protein—but not the bIgG alone—induced significantly higher TNF-α production upon LPS and R848 stimulation. However, the preF protein itself nonsignificantly increased cytokine production as well. This may be explained by its tropism to the insulin-like growth factor receptor 1 (IGFR1), as IGF has been reported to induce innate immune training. Even so, these data suggest a role for IgG-containing ICs in inducing innate immune training after re-exposure to pathogens. However, as ICs of bIgG with a mouse anti-bIgG mAb did not induce this effect, further research is needed to confirm the putative role of bIgG ICs in enhancing innate immune responses in vivo.

## 1. Introduction

Consumption of raw cow’s milk has been shown to be associated with a lower prevalence of asthma and hay fever and even reduced respiratory tract infections in a single study [1,2]. On the other hand, cow’s milk is a major cause of food allergy in approximately 1–4% of very young children [3]. Cow’s milk contains several immunomodulatory proteins that can support the immune system of humans [4,5,6,7]. Bovine IgG or bIgG is one of the major bovine milk proteins that is thought to contribute to the inverse association of raw milk consumption with respiratory tract infections and allergies [8,9,10]. In the gastrointestinal tract or the tonsillar crypts in Waldeyer’s ring, dietary components may come into direct contact with respiratory pathogens from the nasal cavity after swallowing [11,12]. This implies that bIgG can directly encounter bacteria and viruses and form immune complexes (IC). After uptake into the mucosal tissue, these ICs can interact with receptors on immune cells such as neutrophils and macrophages, which phagocytose and eliminate the pathogen. Moreover, upon internalization of ICs, monocytes and dendritic cells (DCs) can process and present antigenic pathogen-derived peptides to T lymphocytes [7,13].

IgG is known to interact with a conserved family of transmembrane glycoproteins known as Fc gamma receptors (FcγRs) [14,15]. On human immune and non-immune cells, three classes of FcγRs are expressed with different affinities for IgG subclasses: high-affinity (10^−9^ M Kd) FcγRI (CD64), and low-affinity (10^−6^ M Kd) FcγRII (CD32) and FcγRIII (CD16) [16,17]. The high-affinity CD64 is predominantly occupied by endogenous serum IgG monomers in vivo [17] and plays a critical role in antibody-dependent cellular phagocytosis (ADCP) in myeloid phagocytes [18]. IgG monomers do not bind to CD32, and only ICs comprising several IgGs bound to antigens can bind to and interact with these low-affinity receptors [19,20]. Along with CD64, CD32 is essential for ADCP by neutrophils and macrophages and also in the process of antigen presentation to the naive T cells by DCs [21,22,23]. The lower affinity of CD32 for IgG monomers ensures that the antibodies’ effector function is only initiated in the presence of a pathogen-derived antigen, preventing an aberrant immune response in the presence of normal levels of antibodies in vivo. CD16, another low-affinity FcγR, is primarily involved in eliminating infected cells via antibody-dependent cellular cytotoxicity (ADCC), mainly mediated by natural killer (NK) cells [21]. Apart from these classical FcγRs, it is known that the neonatal Fc receptor (FcRn) can interact with IgG. FcRn enables the transfer of maternal IgG to the fetus via the placenta, conferring passive immunity to the offspring [24]. FcRn also mediates the salvaging of internalized IgG from degradation through a pH-dependent cellular recycling mechanism [25]. In addition, it was demonstrated that FcRn is important for the internalization of IgG ICs—but not monomers—and the process of antigen presentation by antigen presenting cells (APCs) [26,27].

It has been established that bIgG binds to several human pathogens, including respiratory syncytial virus (RSV) [7,13,28]. Although neutralization of RSV was noted in in vitro studies, the effect of bovine IgG on actual RSV infection that occurs in the nasal cavity cannot be expected; rather, an increased immune response upon re-infection with RSV is anticipated. RSV is one of the most common causes of respiratory tract infections (RTIs) in newborns, which also increases the risk of later-life health complications such as asthma [29,30]. The F protein of RSV is crucial in binding to and infecting human cells, and breast milk preF protein-specific antibodies are a correlate of protection in infants [31]. In addition to binding to and neutralizing human RSV, bIgG facilitates FcγRII-mediated internalization of bIgG-coated pathogens by human neutrophils, monocytes, and DCs [13]. Nederend et al. recently demonstrated that bIgG could neutralize RSV in an in vitro cellular infection model, as did human intravenous immunoglobulin (IVIg) and the prophylactic RSV-specific monoclonal antibody, palivizumab [28]. The latter was more than 100-fold more effective compared to human IV-Ig, and even more compared to bovine IgG, as was expected for a monoclonal antibody. However, as palivizumab is an injection treatment, the clinical efficacy of bIgG cannot be inferred from these findings. Interestingly, the authors showed that activation of RSV preF protein (preF)-specific CD4^+^ and CD8^+^ T cells was strongly enhanced in the presence of bovine IgG [28]. They concluded that the interaction between ICs and (activating) FcγRII on autologous monocytes resulted in higher antigen presentation and T cell activation. Moreover, bIgG was found to be protective against experimental RSV infection in mice [28]. Likewise, dietary supplementation of mice with bovine colostrum, a preparation very rich in bIgG, was shown to decrease RSV infection rates and increase the number of CD69^+^, IFN-γ producing CD8^+^ T cells [32].

Interestingly, bIgG may also contribute to the resistance against (viral) infections by inducing trained innate immunity in FcγR bearing monocytes [33,34]. This mechanism leads to enhanced cytokine production of innate immune cells after stimulation with Toll-like receptor (TLR) ligands [35,36]. In this concept, primary exposure to the training agent leads to a more robust secondary response to the same and related TLR stimulation. The underlying training mechanism for β-glucans—a compound with established training potential—was shown to be via the engagement of the Dectin-1 receptor and downstream signaling events, including the Raf-1 pathway [37,38]. Following activation of the Dectin-1 receptor, epigenetic alterations in the cells occur by trimethylation of the H3K4 histone protein, a shift in cell metabolism from oxidative phosphorylation to aerobic glycolysis, and consequently, a change in the responsiveness of the cells [39,40]. bIgG has been demonstrated to induce innate immune training resulting in increased production of IL-6 and TNF-α in human monocytes in vitro upon TLR stimulation [33,34].

As monomeric IgG does not interact with low-affinity IgG receptors, we hypothesized that the training effects of bIgG might be induced more efficiently by multimeric IgG immune complexes. To address this question, we studied the direct binding of bovine IgG to human monocytes in the presence or absence of the RSV preF protein or anti-bIgG (α-bIgG) antibodies. We established optimal rations between bIgG and the RSV preF and α-bIgG for efficient binding and tested whether these immune complexes could induce innate immune training.

## 2. Materials and Methods

### 2.1. PBMC Isolation

PBMCs were isolated from buffy coats (Sanquin blood bank, Nijmegen) or the fresh blood of donors collected at the Wageningen University blood collection center after obtaining written consent. Gradient centrifugation on Ficoll Paque Plus (GE Healthcare, 17-1440-02, Chicago, IL, USA) was used to isolate PBMCs. Ficoll (15 mL/tube) was transferred to Leucosep tubes (Greiner Bio-One, #227290, Monroe, NC, USA), and the tubes were spun down briefly. Blood samples were added to the tubes after being diluted 1:1 with warm (37 °C) phosphate-buffered saline (PBS) (Gibco, #20012027, Cincinnati, OH, USA). After centrifugation, the PBMCs fraction on top of the porous barrier was transferred to new 50 mL Falcon tubes (Corning, #352070, Corning, NY, USA). Warm PBS was added to wash the cells, and the tubes were spun down. The diluted plasma was discarded, and the cell pellet was resuspended after centrifugation. Following the third wash, the cells were resuspended in RPMI 1640 (Gibco, #61870010, Cincinnati, OH, USA).

### 2.2. Reagents

Bovine Immunoglobulin G (bIgG) was isolated from bovine colostrum and provided by FrieslandCampina. Expression and purification of a DSCav1-like [41] prefusion-stabilized recombinant soluble RSV F protein (preF) were described previously [42]. Monoclonal anti-bovine IgG antibody (α-bIgG) (Sigma-Aldrich, #B6901, St. Louis, MO, USA) was used for bIgG IC formation. For bIgG detection by flow cytometry, AlexaFlour 647 conjugated goat anti-bovine IgG (Jackson ImmunoResearch, #101-605-165, Ely, Cambridgeshire, UK) was applied.

### 2.3. FcγR Expression

The expression of various FcγRs was characterized on different immune cells within the PBMC fraction. PBMCs were stained with fluorochrome-conjugated antibodies (Table 1) for immune cell phenotyping. T- and B-cells, monocytes, mDCs, and pDCs were identified, and the FcγRI (CD64), FcγRII (CD32), and FcγRIII (CD16) expression levels were measured. In brief, 1 × 10^6^ cells were plated in a NUNC plate (ThermoFisher, #267245, Cincinnati, OH, USA) and washed with cold (4 °C) FACS buffer (PBS supplemented with 2.5 mM ethylenediaminetetraacetic acid (EDTA) and 0.05% sodium azide). The cells were then stained with the antibody mixture, and the plate was incubated for 30 min at 4 °C in the dark. Then, the cells were washed with two changes of cold FACS buffer, spinning and discarding the supernatant after each wash. After resuspending the cells in FACS buffer, they were measured on CytoFLEX LX (Beckman Coulter, #C11186, Indianapolis, IN, USA), and the generated data were analyzed using FlowJo (FlowJo LLC, v9, Ashland, OR, USA). The gating strategy for selecting different cell subsets and assessing FcγR expression is described in the supplementary data (Figure S1).

Freshly isolated PBMCs were subjected to various concentrations of bIgG to confirm the binding of bIgG to the monocytes. PBMCs were incubated at 4 °C for 20 min with bIgG (500, 50, 5, and 0 µg/mL) and then were stained with goat AlexaFlour 647 conjugated anti-bovine IgG (Jackson ImmunoResearch, #101-605-165, Ely, Cambridgeshire, UK) and anti-CD14 (Biolegend, #301830, San Diego, CA, USA) for 30 min at 4 °C in the dark. Next, the cells were washed twice with cold FACS buffer. FACS buffer was added, the plate was spun down, and the supernatant was discarded after each centrifugation. The cells were then resuspended in FACS buffer before being analyzed on a CytoFLEX LX flow cytometer. The data were analyzed using FlowJo, and the median fluorescence intensity (MFI) of the bIgG signal was determined on the CD14^+^ cells (Figure S2 for gating strategy).

To determine the optimal antibody: antigen ratio for the formation of large immune complexes (ICs), bIgG was titrated while keeping the concentration of the antigen constant. Increasing concentrations of bIgG were incubated with respiratory syncytial virus (RSV) preF. As the first step and to dispose of antibody aggregates and obtain monomeric forms, the bIgG stock was spun down (17× *g*, RT, 15 min), and the supernatant was used for downstream experiments. bIgG at concentrations of 100, 30, 10, 3, 1, 0.3, 0.1, and 0 µg/mL were made using the serial dilution method and combined 1:1 with PreF protein (50 µg/mL) on a sterile NUNC plate. The plate was wrapped in plastic foil and was pre-incubated at 37 °C for 60 min to allow IC formation. After incubation, the plate was cooled down, and the mixture was exposed to freshly isolated PBMCs (3 × 10^5^/well). The plate was wrapped in foil and was incubated in the fridge (4 °C) for 60 min to allow the binding of ICs to the cells. The cells were washed with cold FACS buffer to remove the unbound antibody/antigen residuals following the incubation. The cells were then stained with anti-bIgG (Jackson ImmunoResearch, #101-605-165, Ely, Cambridgeshire, UK) and also anti-CD14 antibody (Biolegend, #325606, San Diego, CA, USA) for monocyte identification. The same bIgG concentrations but without preF protein (bIgG only) and preF protein alone were included as the experiment controls and background values. Cells were subsequently measured using a CytoFLEX LX flow cytometer, the data were processed using FlowJo, and graphs from the bIgG detection MFI were created using MS Excel (MS Office 365). The experiments were performed with two replicates of the same condition and were repeated with the blood of at least three different donors.

A similar approach was applied to identify the optimal ratio between bIgG and mouse anti-bovine IgG monoclonal antibody (α-bIgG) (Sigma-Aldrich, #B6901, St. Louis, MO, USA). The aim was to use a monoclonal antibody with a higher specificity against bIgG. Various bIgG concentrations (100, 30, 10, 3, 1, 0.3, 0.1, and 0 µg/mL) were incubated with two concentrations of α-bIgG (5 and 1 µg/mL) and the data were handled the same as RSV PreF protein, as described above. The blood samples from at least three donors were used for the titration assays of bIgG and α-bIgG. The optimal antibody: antigen ratio determined in these assays was then utilized in subsequent innate immune training experiments.

### 2.4. Innate Immune Training

The ability of generated ICs to enhance monocyte responses was evaluated in an in vitro innate immune training model [43]. PBMCs were isolated, and CD14^+^ monocytes were negatively selected and trained as described elsewhere [33,34]. RPMI 1640 medium (Gibco, #A1049101, Cincinnati, OH, USA) and 100 µg/mL of whole glucan particle (WGP) (InvivoGen, #tlrl-wgp, San Diego, CA, USA) were applied as the experiment negative and positive controls, respectively. The training was done with preF protein only (50 µg/mL), bIgG only (10 µg/mL), and their corresponding immune complexes (ICs) comprised of bIgG: preF as described earlier. In addition, α-bIgG only (5 µg/mL), bIgG only (3 µg/mL), and bIgG: α-bIgG immune complexes (ICs) were also used separately as the training compounds. After training and resting, the cells were stimulated with either 10 pg/mL of LPS (TLR4 ligand) (Sigma-Aldrich, #L2880, St. Louis, MO, USA) or 5 ng/mL of R848 (TLR7/8 ligand) (InvivoGen, #tlrl-r848, San Diego, CA, USA). Cytometric bead array (CBA) and individual cytokines Flex Sets were used for measuring IL-6 (BD, #558276, Franklin Lakes, NJ, USA) and TNF-α (BD, #558273, Franklin Lakes, NJ, USA) in the culture supernatant of the cells (supplementary Figure S3). The experiments were performed with the PBMCs isolated from 7–10 donors.

### 2.5. Statistical Analysis

CBA data were analyzed by FCAP Array (BD Biosciences, v3.0, Franklin Lakes, NJ, USA) and then were transferred to GraphPad Prism (GraphPad Software, v9, San Diego, CA, USA) for statistical analysis and preparing the figures. The data were normalized and are expressed as fold changes relative to the untrained monocytes (control group-RPMI 1640). The Wilcoxon matched-pairs signed-ranks test was used for head-to-head comparisons, and for multiple comparisons, the Friedman test was utilized to compare different groups with the control. The differences were considered significant when the *p*-value was <0.05 (*), or <0.01 (**), as indicated in the graphs.

## 3. Results

### 3.1. FcγR Expression

To study the relative expression of FcγRs on immune cells, we determined the expression levels of the CD16, CD32, and CD64 on the surface of monocytes, mDC, pDC, and B- and T lymphocytes (Summarized in Table 2). FcγRIII (CD16) was highly expressed on a subset of mDCs (Figure S4A), whereas it was only present on a small percentage (less than 5%) of monocytes, and CD16 was not detected on T- and B cells and on pDCs (Figure S4A). While FcγRII (CD32) expression was high on monocytes and B lymphocytes, this receptor was not present on T cells and pDCs (Figure S4B). CD32 was also present on mDCs; however, the expression levels varied within the mDC subsets (Figure S4B). Monocytes highly expressed FcγRI (CD64); however, in contrast, this receptor was absent on T- and B cells and pDCs (Figure S4C). The expression levels of CD64 on mDCs varied considerably (Figure S4C).

**Figure 1:**
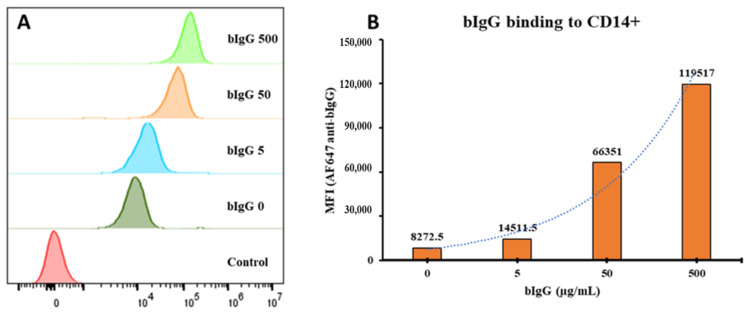
Histogram comparing the mean fluorescence intensity (MFI) of the bovine IgG (bIgG) signal on CD14^+^ monocytes: Peripheral blood mononuclear cells (PBMCs) were incubated with bIgG (500, 50, 5, or 0 µg/mL) for 20 min (**A**). A dose-dependent increase in the bIgG signal on the CD14^+^ monocytes was detected with an increase in the concentration of bIgG used (**B**).

Although indirect immunological effects of bIgG on monocytes and other cell types was described previously [14], the direct binding of bIgG to monocytes has not formally been demonstrated. To demonstrate the binding of bovine IgG to human monocytes, a range of bIgG concentrations was allowed to bind to human PBMCs, washed to remove non-bound bIgG, and binding to monocytes was detected by flow cytometry using AlexaFlour 647-conjugated anti-bovine IgG antibody. Monocytes were selected since they highly express CD32, the same FcγR that bIgG was shown to bind [13]. Bovine IgG showed a dose-dependent binding to human monocytes, especially at high bIgG concentrations (Figure 1A,B). As we had previously noted the presence of some aggregated bIgG on native-PAGE, this binding at high bIgG concentrations might be related to the aggregated bIgG. The presence of bIgG aggregates before centrifugation and their removal by centrifugation is shown in Figure S5. In this figure, the apparent aggregates of IgG are clearly visible in the starting material as in the pellet, but not in the supernatant after centrifugation. Although these data are not quantitative, the difference between the supernatant and pellet fraction indicate the presence of aggregates, as well as the fact that they are (at least partially) depleted by centrifugation prior to use.

**Figure 2:**
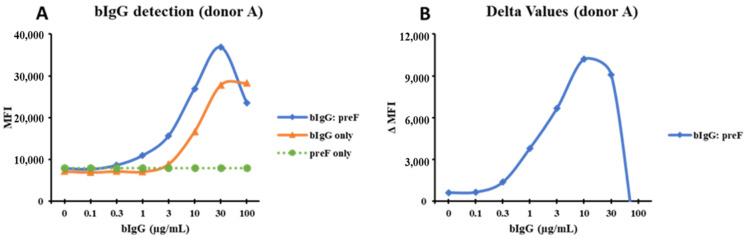
The curves drawn from MFI of the bIgG detection signal: CD14^+^ monocytes were exposed to bIgG only (0–100 µg/mL), preF protein only (50 µg/mL), and the bIgG: preF ICs in one representative donor (donor A); the MFI was used to generate the detection curve (**A**). The delta MFI (ΔMFI) was produced when the MFI of the bIgG only signal was deducted from the MFI of the bIgG: preF ICs signal (**B**).

To study if the binding to FcγRs on monocytes is increased by immune complexes, various concentrations of bIgG were preincubated with the RSV preF protein or with a monoclonal anti-bIgG to allow the formation of ICs before exposing them to the PBMCs. As shown in Figure 2A, we noted a dose-dependent binding of bIgG alone to the CD14^+^ monocytes. However, the combination of bIgG and preF protein (bIgG: preF) showed a higher binding, especially at lower bIgG concentrations used, suggesting that multivalent ICs are formed and bound to the monocytes (Figure 2A). Subtraction of the bIgG signal from the MFI of the bIgG: preF combination (ΔMFI) resulted in a bell-shaped curve suggestive of immune complex binding (Figure 2B). The ΔMFI curve had a peak at 10 µg/mL and 50 µg/mL for bIgG and PreF protein, respectively, indicative of the optimal ratio for the formation of large ICs. We observed comparable findings for additional donors tested, with the bell-shaped curves peaking at 10 µg/mL of bIgG (Figure S6A–D) and the average maximum ΔMFI of about 17k B and Figure S6B,D). The data obtained for the three different donors were thus highly consistent.

**Figure 3:**
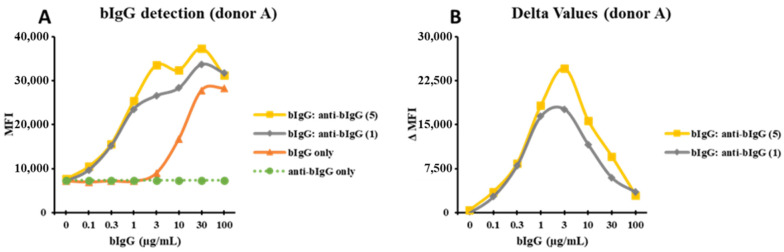
The curves drawn from MFI of the bIgG detection signal: the monocytes were exposed to bIgG only (0–100 µg/mL), α-bIgG (5 µg/mL) only, α-bIgG only (1 µg/mL), and the bIgG: α-bIgG ICs in one representative donor (donor A) (**A**). The delta MFI (ΔMFI) was produced when the MFI of the bIgG only signal was deducted from the MFI of the bIgG: α-bIgG ICs signal (**B**).

As the window between the binding of bIgG: preF ICs and bIgG alone was relatively small, we tried a similar approach by using a monoclonal anti-bIgG (α-bIgG) in the hope that because of the high specificity, the peak of the binding would be at a lower bIgG concentration with even lower bIgG background binding. bIgG bound to human monocytes in a dose-dependent manner, while α-bIgG alone did not show any binding (Figure 3A). However, the combination of bIgG and α-bIgG showed a strong increase in binding to the monocytes suggesting IC formation (Figure 3A). This was true for both α-bIgG concentrations that were used (5 and 1 µg/mL). After subtracting the MFI of bIgG alone from the MFI of the ICs, we obtained bell-shaped curves with a peak at bIgG 3 µg/mL (Figure 3B). Similar results were found for additional donors with the bell-shaped curves, although these donors peaked at a slightly lower concentration of 1 µg/mL of bIgG (Figure S7A–D) and the average maximum ΔMFI of about 40k (Figure 3B and Figure S7B,D). As the optimal concentrations for the formation of large ICs are at 3 and 5 µg/mL for bIgG and α-bIgG antibody, respectively, these concentrations were used in innate immune training experiments.

### 3.2. BIgG-Containing Immune Complexes and Innate Immune Training

Based on the above findings, we used the optimal ratios of bovine IgG to RSV preF protein or α-bIgG to study if these immune complexes could induce innate immune training in vitro at concentrations at which bIgG itself had no effect in this model. The generated ICs were allowed to bind to freshly isolated CD14^+^ monocytes for 24 hrs, after which the training compounds were removed by washing and resting for 6 days. After this resting period, the cells were stimulated with TLR4 (LPS) or TLR7/8 (R848) ligands. Then, 24-h supernatants were collected, and the levels of IL-6 and TNF-α were measured by CBA.

**Figure 4:**
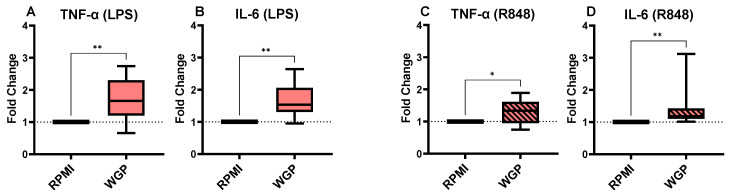
The fold changes in TNF-α (**A**) and IL-6 (**B**) production of the cells trained with WGP and stimulated with LPS in comparison to the untrained control. The TNF-α (**C**) and IL-6 (**D**) production fold changes for WGP-trained monocytes after stimulation with R848. The average (range) of cytokines in the RPMI controls of all 8 donors tested were 1805 (719–4825 pg/mL), 3181 (1044–7232 pg/mL), 3592 (1839–7434 pg/mL), and 9047 (3732–14230 pg/mL) for conditions A to D, respectively. The boxes represent 50% of the data, and the line is the median value where upper and lower whiskers present the upper and lower 25% of the data, respectively. The significance of differences is shown as *p*-value < 0.05 (*) and < 0.01 (**).

Whole glucan particles (WGP) were used as a positive control for training, as described by Moerings et al. [44]. The cells trained with WGP produced a significantly higher amount of IL-6 and TNF-α in the culture supernatant upon re-stimulation with LPS (Figure 4A,B). We obtained similar results when stimulating the cells with R848, and the cells trained with WGP produced significantly more IL-6 (Figure 4C) and TNF-α (Figure 4D) than untrained cells.

**Figure 5:**
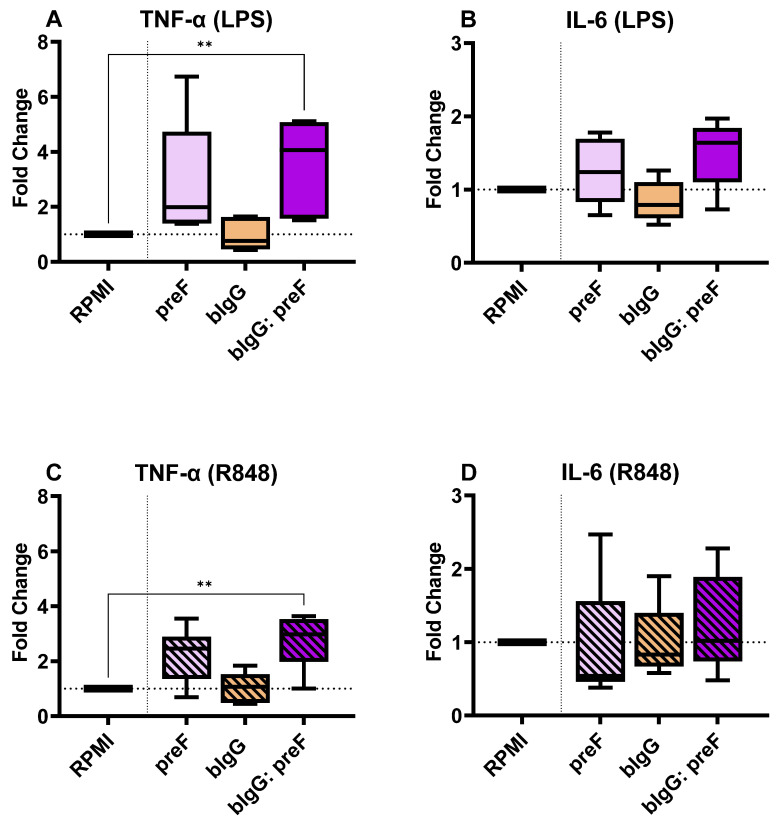
The fold changes in the TNF-α (**A**) and IL-6 (**B**) production in the cells trained with preF only (50 µg/mL) bIgG alone (10 µg/mL), or bIgG: preF ICs compared to untrained monocytes (RPMI) upon stimulation with LPS. Also, the TNF-α (**C**) and IL-6 (**D**) production fold changes compared to the RPMI control in the group of monocytes trained with single components or the bIgG: preF ICs after stimulation with and R848. The average (range) of cytokines in the RPMI controls of all 8 donors tested were 1791 (719–4825 pg/mL), 3522 (1044–7232 pg/mL), 3181 (1839–7434 pg/mL), and 9840 (3732–14,230 pg/mL) for conditions A to D, respectively. The boxes represent 50% of the data, and the line is the median value where upper and lower whiskers present the upper and lower 25% of the data, respectively. The significance of differences is shown as *p*-value < 0.01 (**).

Similarly, IL-6 and TNF-α production levels were determined in the supernatants of restimulated monocytes that were trained with RSV preF protein only (50 µg/mL), bIgG only (10 µg/mL), or the ICs of the combination of these. The cells trained with bIgG: preF ICs showed significantly higher (3–4-fold) TNF-α levels compared to the untrained cells, both after stimulation with LPS (Figure 5A) and R848 (Figure 5C). TNF-α levels were not significantly increased in the cells trained with RSV preF protein only or bIgG alone, although some increase was seen in the preF protein group. Despite variation in the responses, all donors exposed to the ICs consistently produced higher levels of TNF-α compared to the untrained monocytes. Even though IL-6 levels were slightly increased after stimulation with the TLR4 ligand LPS (Figure 5B) or TLR7/8 ligand R848 (Figure 5D), this did not reach significance.

**Figure 6:**
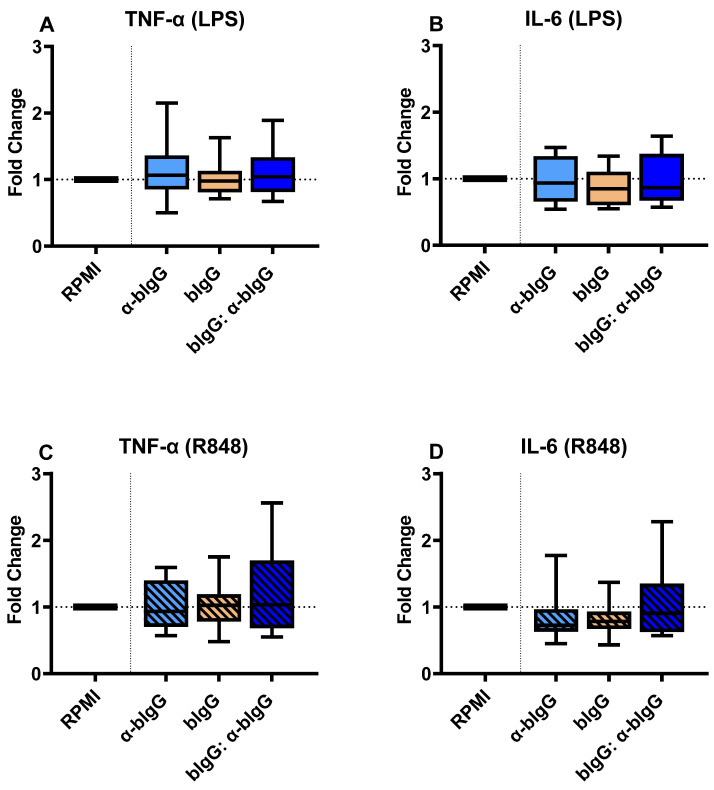
The fold changes in the production of TNF-α (**A**) and IL-6 (**B**) in the cells trained with α-bIgG only (5 µg/mL) only, bIgG alone (3 µg/mL), or bIgG: α-bIgG ICs compared to untrained monocytes (RPMI) upon stimulation with LPS and also TNF-α (**C**) and IL-6 (**D**) production after R848 stimulation. The average (range) of cytokines in the RPMI controls of all 8 donors tested were 1740 (719–4825 pg/mL), 3279 (1044–7232 pg/mL), 3508 (1839–7434 pg/mL), and 8961 (3732–14,230 pg/mL) for conditions A to D, respectively. The boxes represent 50% of the data, and the line is the median value where upper and lower whiskers present the upper and lower 25% of the data, respectively.

The same experimental setup was performed with bIgG: anti-bIgG ICs, evaluating the training potential of monoclonal anti-bIgG alone (5 µg/mL), bIgG alone (3 µg/mL), and the ICs resulted from combining both. As shown in Figure 6, contrary to the bIgG-RSV preF protein IC, no increases in IL-6 and TNF-a were noted. This holds true for stimulation with either LPS (Figure 6A) or R848 (Figure 6C). Similar to TNF-α, we did not observe any significant variation in the production of IL-6 in different conditions. Neither α-bIgG only, bIgG only, nor the ICs could enhance IL-6 production in monocytes after LPS (Figure 6B) or R848 (Figure 6D) stimulation.

## 4. Discussions

Here, we show that immune complexes comprising bIgG and RSV preF protein can induce innate immune training in human CD14^+^ monocytes, while bIgG monomers did not have the same effects. We established and optimized an experimental system for detecting the direct binding of ICs to human monocytes. Using that system, we determined the optimal antibody: antigen ratio for the formation of ICs between bIgG and RSV preF protein, as well as bIgG: α-bIgG, and tested them on the in vitro innate immune training model.

The FcγRII (CD32) family consisting of CD32a, CD32b, and CD32c are key IgG receptors expressed by various leukocytes. While CD32a and CD32c have a tyrosine-based activation motif (ITAM) on their C-terminal cytoplasmic tail mediating the activator signal, CD32b contains a tyrosine-based inhibitory motif (ITIM) [23]. CD32 expression has been identified in monocytes, DCs, and B cells, as well as neutrophils, eosinophils, basophils, and mast cells [14,23]. Monocytes/macrophages and DCs eminently express CD32a with an ITAM motif, but also CD32b, while B cells exclusively possess the inhibitory CD32b [23]. The interplay between the activator and inhibitory signal regulates the antibody effector functions, including B cell IgG responses, APC maturation, and antigen presentation [45,46]. CD32 expression on monocytes and DCs is essential for their distinct functions, including ADCP for clearing the pathogens, also known as FcγRII-dependent phagocytosis [47,48]. Following the phagocytosis of antibody-opsonized targets, the capacity of DCs for activating naive T cells increases [49]. DCs present the antigen epitopes on major histocompatibility complex class II (MHC-II) molecules, upregulate the expression of costimulatory molecules such as CD80/86 to interact with their T cells counterpart (CD28), and produce cytokines to drive T cell differentiation [50]. However, monomeric forms of IgG do not bind to low-affinity receptors such as CD32. Binding to and crosslinking multiple neighboring CD32 is essential for both FcγR-mediated phagocytosis and initiating the signal via the receptors’ ITAM motif [51]. In fact, signaling cascades are activated by immunoreceptor aggregation rather than ligand-induced changes in receptor conformation [51]. In other words, the size matters when it comes to the level of IC binding and interaction with the low-affinity FcγRs, and only large multivalent ICs can bind to CD32 and induce the effector function [20,52]. Large ICs can be formed when an optimal antibody: antigen ratio is present in the environment. When either the antibody or the antigen is in excess, large ICs are not formed, the interaction with CD32 does not occur, and hence, the antibody’s effector function is weak [14]. As a result, the concentration-dependent binding of immune complexes to receptors typically results in a bell shaped curve, as confirmed in this paper.

RSV-specific IgG in breast milk was shown to correlate with protection against RSV acute respiratory infection in the first 6 months of life [31]. Bovine milk IgG (bIgG) binds to and can neutralize RSV, a major human pathogen associated with respiratory tract infections, in in vitro infection studies [13]. In addition, bIgG was shown to interact with the FcγRII on human immune cells, which is essential for exerting the antibody effector functions [13,28]. The interaction of bIgG with human immune cells conferred protection against experimental RSV infection in mice and also increased activation of RSV-specific human T cells [28]. The augmented T cell activity at low RSV preF protein levels is an effect resulting from the direct interaction of antigen-bound bIgG (ICs) with APCs. The interaction of IC with APCs enhances antigen presentation to RSV-specific T cells. However, the direct binding of bIgG ICs to APCs has not been shown before. In the current study, we succeeded in generating bIgG and RSV preF protein ICs and demonstrated their direct binding to CD14^+^ monocytes (Figure 2). The bell-shaped curves on ΔMFI results indicate that large ICs were formed and bound to CD32 on monocytes. The findings are especially important since we show the binding of bIgG ICs to the APC, an intermediate step linking the previously shown bIgG and preF binding with the consequent enhanced T cell response.

The method described here can be applied in future studies as a proxy to identify the optimal bIgG: antigen ratio for IC formation for additional pathogenic molecules, and can also be used to detect IC binding on other immune cells, such as neutrophils and DCs. We selected monocytes to study bIgG ICs binding for a number of reasons. Monocytes and B cells both express a high level of CD32 and the results from CD19^+^ B cells support the findings from CD14^+^ cells on optimal bIgG: antigen ratios (data not shown). However, unlike B cells that only express inhibitory CD32b, monocytes eminently express CD32a, which is essential for studying the cell-activating properties of bIgG ICs. This includes the innate immune training model applied in this research, which also has been optimized for monocytes.

It should be noted that the study did not address the relevance of the FcRn receptor in bIgG binding to monocytes because the binding of bovine IgG to human monocytes was reported to be mainly CD32-dependent [13]. However, FcRn is expressed on monocytes, macrophages, and DCs [53], and its role in IgG IC-mediated antigen presentation has been demonstrated [26,27]. As blocking with anti-CD32/CD16 antibodies [13] or anti-CD16/32/64 [54] cannot completely block the binding of bIgG to monocytes and granulocytes, a role for FcRn cannot be excluded.

Concurrent engagement and crosstalk between FcγRs and pattern recognition receptors (PRRs) are critical for identifying and eliminating the pathogen [55,56]. TLRs and C-type lectin receptors (CLRs) are among the PRRs that were found to be involved in this crosstalk, which is necessary for the induction of inflammatory mediators such as IL-6 and TNF-α [55,56]. On the other hand, trained immunity is mediated by the involvement of CLRs, such as Dectin-1, as was demonstrated for β-1, 3-(D)-glucan derived from *Candida albicans* [37]. After training, the quality of cell responses improves, as evidenced by increased IL-6 and TNF-α production in response to TLR re-stimulation [57]. This trained immunity results in enhanced innate immune responses to a wide array of TLR signals, resulting in improved protection against infection. Given that bIgG has been demonstrated to possess training abilities and interact with FcγRs, we hypothesized that these receptors would play a role in monocyte training. Immune complexes internalized via the engagement of FcγRs can stimulate endosomal or cytoplasmic PRRs such as TLRs to further activate the cells [58,59,60,61,62]. If the assumption holds true, ICs may be more potent training-inducing components than bIgG alone.

Although the training effects of bIgG have been demonstrated previously [33,34], the relevance of ICs in the training effects has not been studied. CD64 receptors on monocytes freshly isolated from human blood are occupied by human serum IgG, leaving no room for bIgG monomers to bind. Monomeric forms of bIgG, on the other hand, do not interact with the low-affinity CD32. Therefore, if we assume that the training potential of bIgG is (partly) exerted via interacting with FcγRs, the engagement of bIgG ICs and CD32 is critical. The ICs can crosslink several receptors and induce a much stronger effector signal. Surprisingly, although no antigen was added to the bIgG preparations and, therefore, no ICs are expected, the training effects were still evident in the previous studies [33,34]. The explanation could be within the bIgG stock itself. IgG molecules tend towards aggregation, particularly after (long) storage in the freezer [63]. It is likely that the antibody aggregates present in the bIgG stock have mimicked IC properties and could be responsible for the previously observed training effects of bIgG alone, especially since they used high concentrations of the IgG.

We exposed monocytes to bIgG monomers and bIgG: preF ICs as the training agents to address this hypothesis. The experiment’s positive control, WGP (a Dectin-1 agonist with an established training potential [44]), ensured the validity of the model system used. Interestingly, incubation with the ICs increased the TNF-α production in the cells upon TLR stimulation (Figure 5). Pair-wise comparisons showed that IC-trained cells produced 2–4 times higher TNF-α than the untrained cells upon LPS and R848 stimulation. Remarkably, the training effect was not seen for the monomeric forms of the bIgG. This is in line with the fact that the bIgG alone concentration used is too low to induce innate immune training.

Given the increased TNF-α response, it appears that there is a general increase in the vigilance of the monocytes. The heightened response was observed towards not only TLR7/8 activation with R848, but also LPS. The ICs contained RSV preF protein as a viral protein. Interestingly, higher TNF-α levels were also produced by IC-trained cells after stimulation of TLR4 with LPS, a compound found in the membrane of Gram-negative bacteria. A documented aspect of trained immunity is an increase in the responsiveness of the cells to the same but also homologous stimuli [64]. Nevertheless, further research on cells’ epigenetic changes and metabolic pathways is required to substantiate this notion.

The monocytes trained with bIgG: preF ICs also produced a relatively higher IL-6 than the untrained cells in response to LPS stimulation (Figure 5B). However, the changes did not reach statistical significance, probably due to higher variation in the response of the donors. In fact, TNF-α was previously described as a better indicator of innate immune training by bIgG than IL-6 [34]. However, the RSV preF protein alone seemed to induce some increase in the production of TNF-a and IL-6 in these assays, although this did not reach significance. Interestingly, the preF protein was recently shown to bind to insulin-like growth factor 1 (IGFR1) for cellular entry [65]. IGF, the natural ligand for this receptor, has been shown to induce innate immune training [66], which can explain why the preF protein by itself has an effect on this model system as well. In addition, viruses and viral proteins may be internalized, given their antigenic nature, without the involvement of antibodies. When they are internalized, they can activate cytoplasmic or endosomal PRRs, inducing inflammatory responses. This fact could also partly explain why monocytes treated with RSV preF alone released more cytokines. More investigation is needed to determine what we described is the reason or whether a contaminant in the preparation caused the effect.

Contrary to our expectations, incubation with IC consisting of bIgG with a murine α-bIgG monoclonal antibody (mAb) did not induce monocyte training (Figure 6). The optimal ratio for bIgG and the α-bIgG antibody to form ICs was identified, and even the average of maximum ΔMFI for the α-bIgG ICs on the three donors tested was more than twice the level of the preF ICs (40k vs. 17k). This difference, in theory, should give a bigger window for the effects of α-bIgG ICs than preF ICs, which was not the case in practice. A possible explanation of these findings is that lower bIgG concentrations were used to generate ICs with α-bIgG than RSV preF protein (3 vs. 10 ug/mL). In addition, it is known that murine IgG does not bind efficiently to human CD32 [19]. This could result in ICs that consist of bIgG, of which the Fc region is mostly blocked by the murine mAb. As a result, in α-bIgG ICs, fewer bIgG molecules may be available to interact with CD32 on the monocytes, which may not result in efficient crosslinking and uptake of the IC. As the binding of the IC was detected with a polyclonal anti-bIgG, higher levels of binding were detected in the FACS analysis of these IC. Another highly likely explanation for no training effects could be the absence of the antigen. IgG bound to antigen likely sends a different signal via FcγRs than an antibody linked to another antibody, possibly due to different IgG Fc glycosylation patterns between the different antibodies used [16,67]. In addition, when the ICs that contain antigens are internalized, other endosomal or cytoplasmic PRRs, such as CLRs and TLRs, may become activated and synergistically complement the FcγRs signal, as discussed earlier. However, further investigation is necessary before ascertaining these claims.

In conclusion, we established a method for detecting the direct binding of bIgG-containing ICs to monocytes by flow cytometry. Our results also indicate that bIgG: preF ICs can induce monocyte training in vitro. The effects could be at least partly mediated by the interaction of bIgG ICs with the CD32 receptors on the monocytes, as this interaction was shown before. However, to formally prove the putative role of bIgG ICs in trained immunity, this has to be investigated with more antigen-bIgG ICs and should be extended to also include human IgG-antigen ICs.

## Figures and Tables

**Table 1:** Antibody panel used for peripheral blood mononuclear cell (PBMC) phenotyping and assessing the expression of FcγRs.

Antibody	Fluorochrome	Host/Isotype	Clone	Company	Catalog Number
α-CD3	PE-Cy5	Mouse/ IgG1	UCHT1	BD	555,334
α-CD11c	BV421	Mouse/IgG1	3.9	Biolegend	301,628
α-CD14	APC-H7	mouse/IgG2b	MφP9	BD	560,180
α-CD19	FITC	Mouse/IgG1	HIB19	BD	555,412
α-CD123	BV605	mouse/IgG2a	7G3	BD	564,197
α-HLA-DR	BV510	mouse/IgG2a	L243	Biolegend	307,646
α-CD64	APC	mouse/IgG1	10.1	Biolegend	305,014
α-CD32	PerCp-Cy5.5	mouse/IgG2b	FUN-2	Biolegend	303,216
α-CD16	PE	mouse/IgG1	B73.1	BD	332,779

Detection of bIgG and bIgG-immune complexes bound to monocytes.

**Table 2:** Direct binding of bIgG and bIgG-immune complexes to monocytes.

Cell Type	FcγRIII (CD16)	FcγRII (CD32)	FcγRI (CD64)
T cells	−	−	−
B cells	−	+	−
Monocytes	+/−	+	+
mDCs	+/−	+	(+)
pDCs	−	−	−

+, the receptor is expressed on the cell; −, the receptor is not expressed on the cell; +/−, the receptor is expressed on a subset of the cell; (+), different levels of receptor expression.

## Data Availability

The data presented in this study are available on request from the corresponding author.

## Supplementary Materials

The following supporting information can be downloaded at: https://www.mdpi.com/article/10.3390/nu14214452/s1. Figure S1. The gating strategy to identify T- and B cells, monocytes, mDC, and pDCs. Afterward, CD16, CD32, and CD64 expression levels were determined within each cell population. Figure S2. The gating strategy for selecting the CD14^+^ monocytes and quantifying the MFI of the bIgG signal. Figure S3. A schematic representation of the in vitro innate immune training model. CD14^+^ monocytes were isolated from the buffy coats, exposed to the training compounds for 24 h, and rested for six days to differentiate into macrophages. Then the cells were stimulated for 24 h with TLR ligands (LPS and R848), and the production of IL-6 and TNF-α was quantified in the culture supernatant of the cells. Figure S4. Histograms showing the relative expression of CD16 (A), CD32 (B), and CD64 (C) on T- and B lymphocytes, monocytes, mDCs, and pDCs cells within the PBMC fraction. See Supplementary Figure S1 regarding the gating strategy for PBMC immunophenotyping. Figure S5. Native-PAGE gel from bIgG samples. The bIgG stock was spun down to remove the aggregates. Different concentrations (1000, 100, 10 µg/mL) of the stock itself, the supernatant after centrifugation, and the pellet were prepared in a non-reducing non-denaturing sample buffer (without SDS) and were loaded on the 4-15% 4–15% Mini-PROTEAN TGX Precast Protein Gel (Bio-RAD #456-1083). The gel was run at 110v for 75 min. The silver staining was used to stain the gel. Figure S6. The curves drawn from MFI of the bIgG detection signal: The generation of bIgG detection curve for bIgG only, preF protein only, and the bIgG: preF ICs on monocytes of two selected donors (A & C). The delta MFI values resulted from the deduction of bIgG background values from the ICs curve (B & D). Figure S7. The curves drawn from MFI of the bIgG detection signal: bIgG detection curves were generated for bIgG only, α-bIgG (5 µg/mL) only, α-bIgG (1 µg/mL) only, and the ICs on monocytes of two selected donors (A & C). The delta MFI values resulted from the deduction of bIgG background values from the ICs curve (B & D).
