# Supplementary material for: Direct Binding of Bovine IgG-Containing Immune Complexes to Human Monocytes and Their Putative Role in Innate Immune Training
Source: Nutrients. 2022 Oct 22;14(21):4452. doi: 10.3390/nu14214452 (PMC9654672; doi:10.3390/nu14214452)
Supplement: Supplementary file 1 [file nutrients-14-04452-s001.zip › nutrients-1898723-supplementary.pdf]

Supplementary data

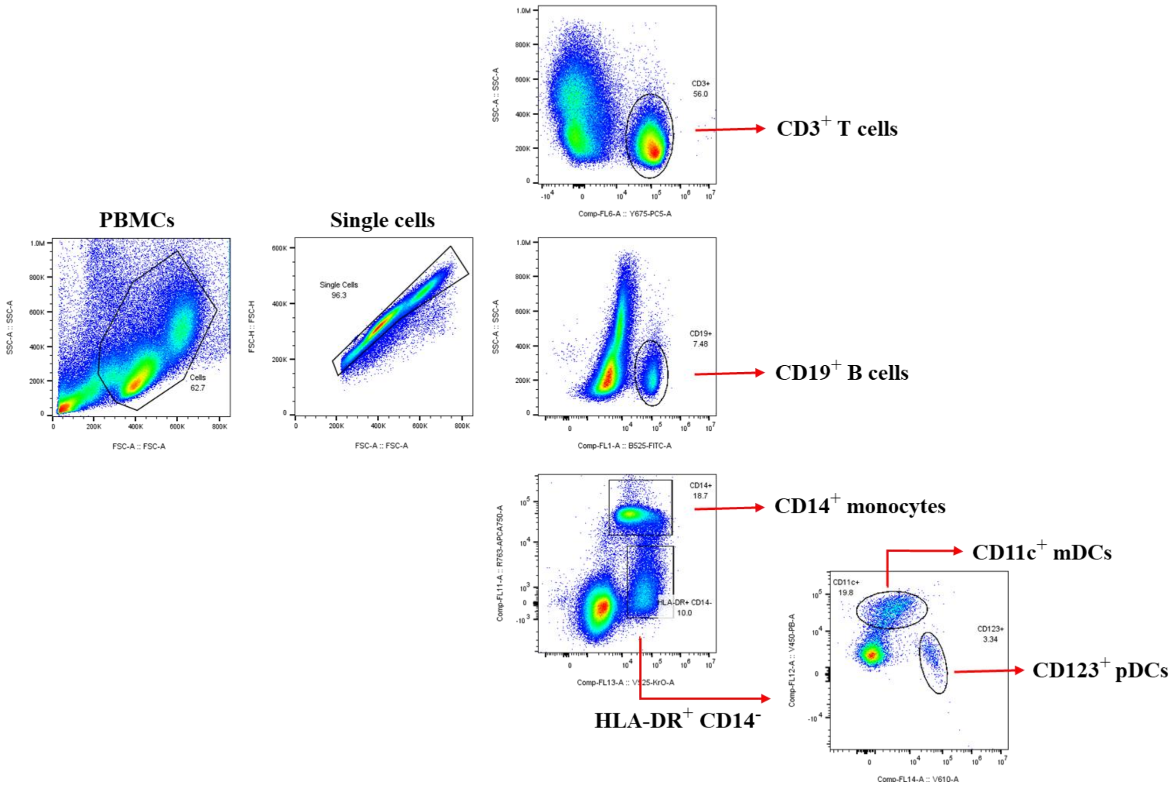

Figure S1. The gating strategy to identify T- and B cells, monocytes, mDC, and pDCs. Afterward, CD16, CD32, and CD64 expression levels were determined within each cell population.

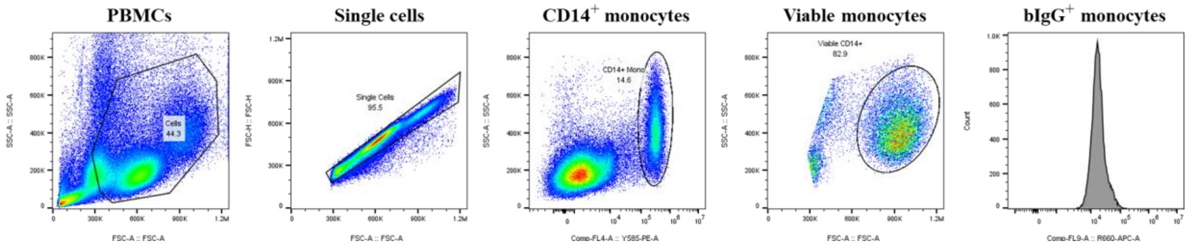

Figure S2. The gating strategy for selecting the CD14⁺ monocytes and quantifying the MFI of the bIgG signal.

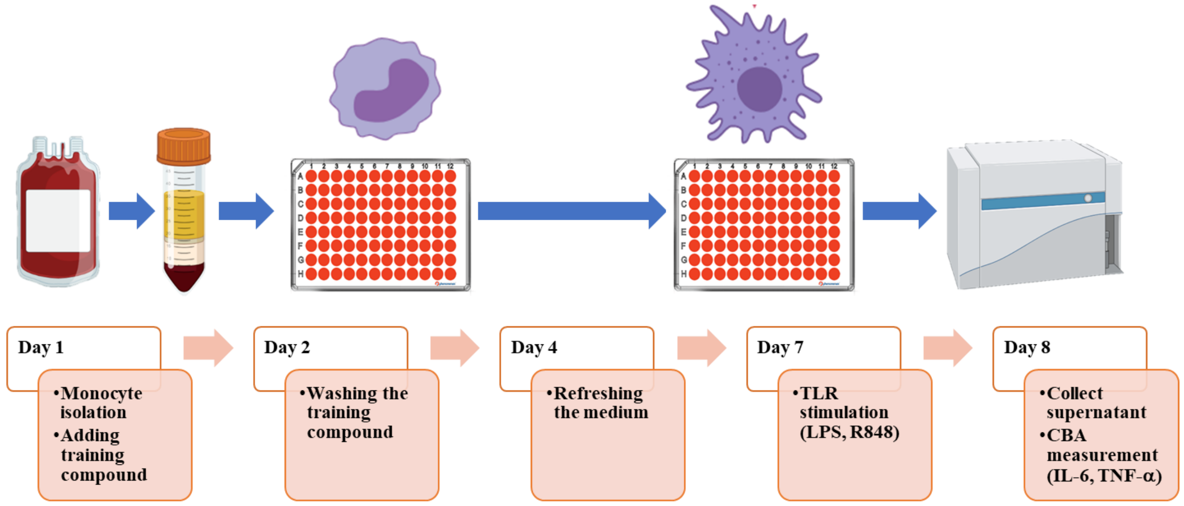

Figure S3. A schematic representation of the in vitro innate immune training model. CD14+ monocytes were isolated from the buffy coats, exposed to the training compounds for 24 hours, and rested for six days to differentiate into macrophages. Then the cells were stimulated for 24 hours with TLR ligands (LPS and R848), and the production of IL-6 and TNF-α was quantified in the culture supernatant of the cells.

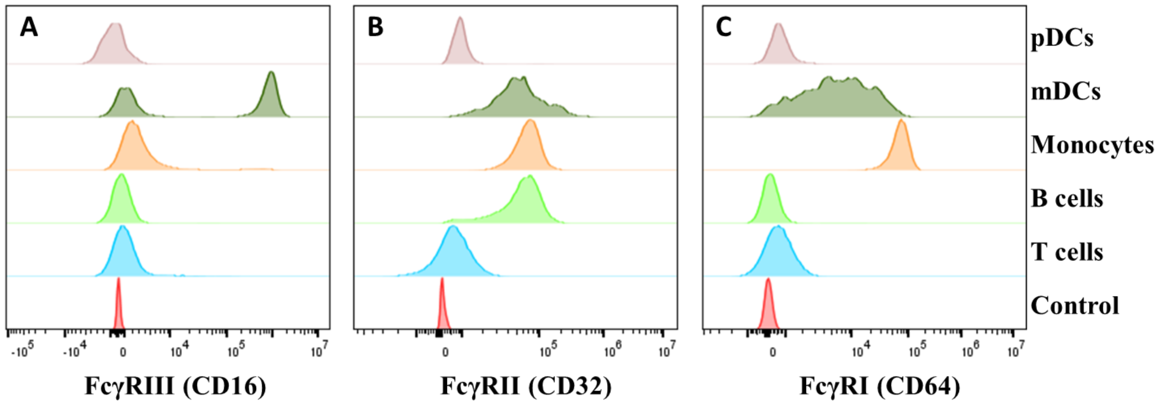

Figure S4. Histograms showing the relative expression of CD16 (A), CD32 (B), and CD64 (C) on T- and B lymphocytes, monocytes, mDCs, and pDCs cells within the PBMC fraction. See Supplementary Fig. S1 regarding the gating strategy for PBMC immunophenotyping.

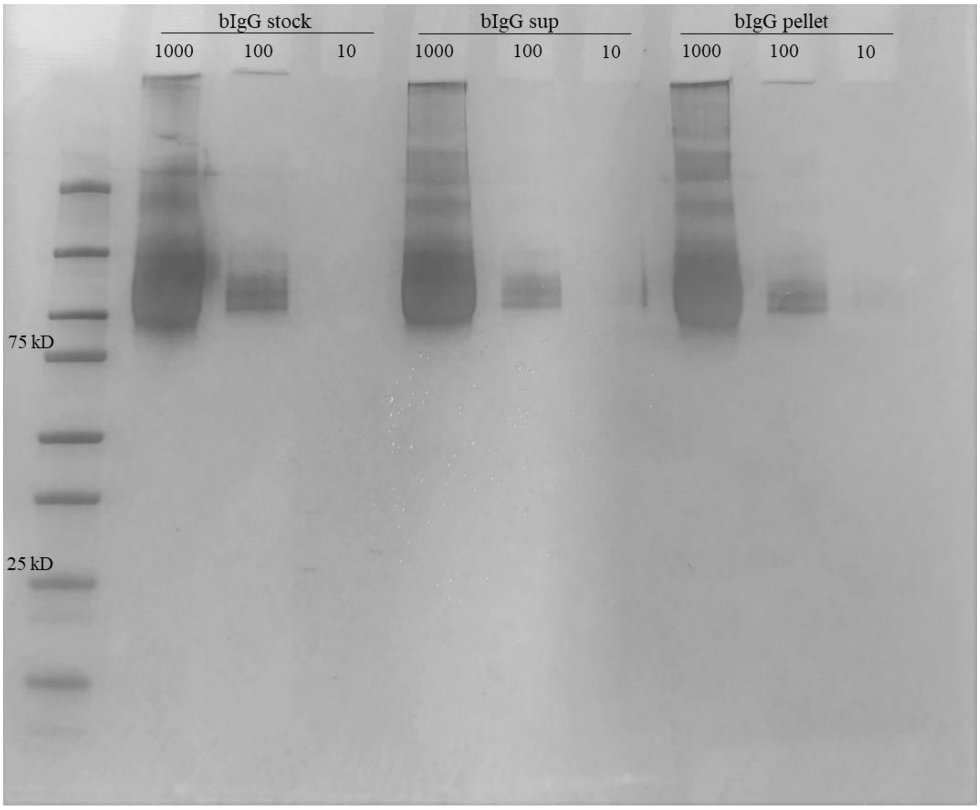

Figure S5. Native-PAGE gel from bIgG samples. The bIgG stock was spun down to remove the aggregates. Different concentrations (1000, 100, 10 µg/mL) of the stock itself, the supernatant after centrifugation, and the pellet were prepared in a non-reducing non-denaturing sample buffer (without SDS) and were loaded on the 4-15% 4–15% Mini-PROTEAN TGX Precast Protein Gel (Bio-RAD #456-1083). The gel was run at 110v for 75 min. The silver staining was used to stain the gel.

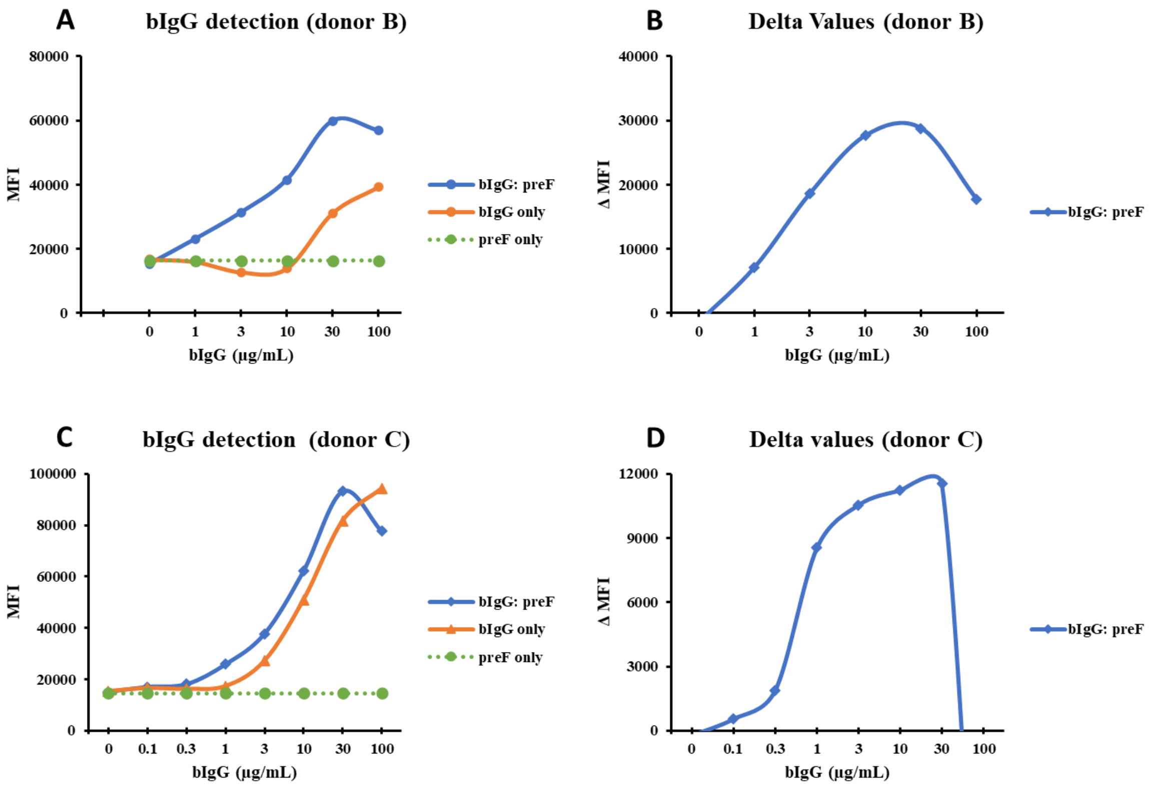

Figure S6. The curves drawn from MFI of the bIgG detection signal: The generation of bIgG detection curve for bIgG only, preF protein only, and the bIgG: preF ICs on monocytes of two selected donors (A & C). The delta MFI values resulted from the deduction of bIgG background values from the ICs curve (B & D).

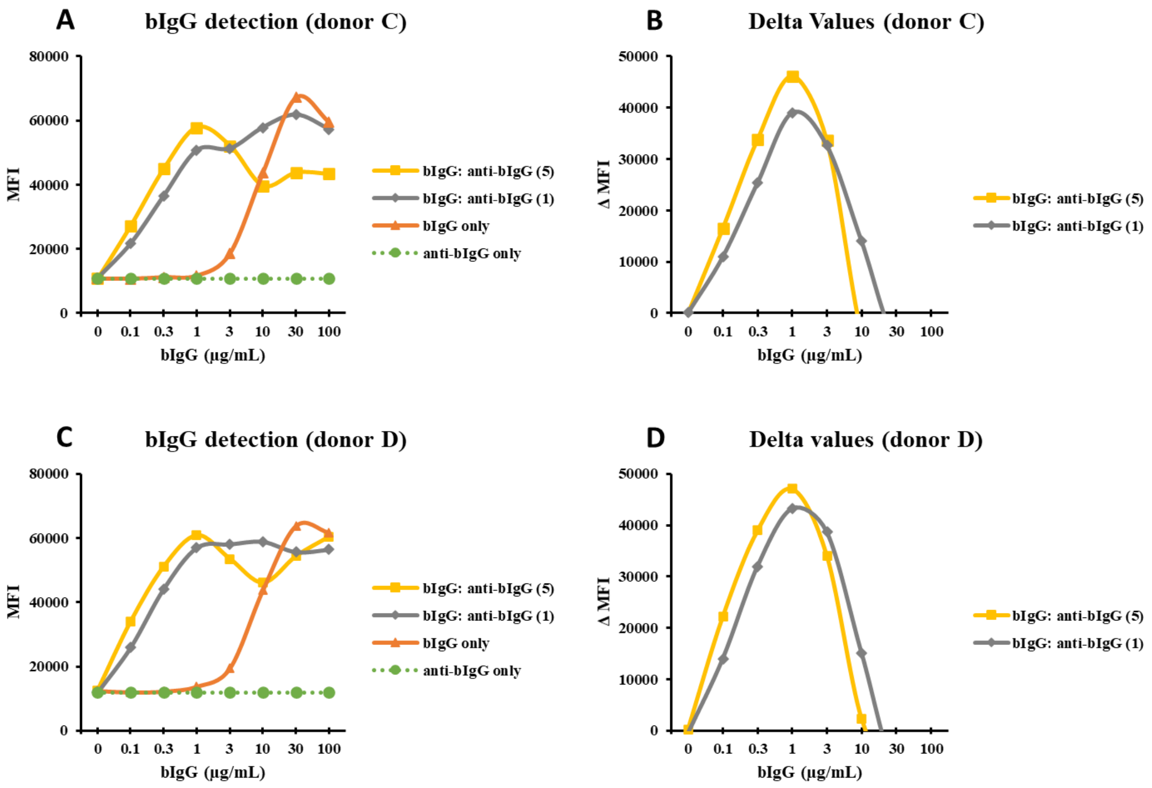

Figure S7. The curves drawn from MFI of the bIgG detection signal: bIgG detection curves were generated for bIgG only, α-bIgG (5 μg/mL) only, α-bIgG (1 μg/mL) only, and the ICs on monocytes of two selected donors (A & C). The delta MFI values resulted from the deduction of bIgG background values from the ICs curve (B & D).
